# Combined Cognitive and Motor Training Improves Reading, Writing and Motor Coordination in Dyslexic Children

**DOI:** 10.3390/pediatric17020046

**Published:** 2025-04-10

**Authors:** Amal Ben Dhia, Maria-Pia Bucci, Chokri Naffeti, Helmi Ben Saad, Omar Hammouda, Tarak Driss

**Affiliations:** 1 Interdisciplinary Laboratory in Neurosciences, Physiology and Psychology: Physical Activity, Health and Learning (LINP2), UFR STAPS (Faculty of Sport Sciences), Paris Nanterre University, 92000 Nanterre, France; bendhiaamal@gmail.com (A.B.D.); omar.hammouda@parisnanterre.fr (O.H.); 2 ICAR UMR 5191 CNRS, ENS de Lyon, Université Lyon 2, 15, Parvis René Descartes, 69342 Lyon, France; mariapia.bucci@gmail.com; 3 Equipe InDev (NeuroDiderot), Hôpital Universitaire Robert Debré, Consultation ORL, 48 Bd Sérurier, 75019 Paris, France; 4 Research Laboratory Education, Motricité, Sport et Santé, EM2S, LR19JS01, High Institute of Sport and Physical Education of Sfax, University of Sfax, Sfax 3000, Tunisia; chokri.judo@yahoo.fr; 5 Heart Failure (LR12SP09) Research Laboratory, Farhat Hached Hospital, Faculty of Medicine of Sousse, University of Sousse, Sousse 4002, Tunisia; helmi.bensaad@rns.tn; 6 Research Laboratory, Molecular Bases of Human Pathology, LR19ES13, Faculty of Medicine, University of Sfax, Sfax 3000, Tunisia

**Keywords:** dyslexic children, combined cognitive and motor training, motor abilities, reading and writing capabilities

## Abstract

Background/Objectives: Different strategies were proposed to enhance dyslexic children’s performance. This study aimed to investigate the effect of combined cognitive and motor training on reading, writing, and motor coordination in dyslexic children. Methods: Twenty-four children with dyslexia (9.33 ± 0.48 years) were randomly allocated to either a Control (CG, 9.25 ± 0.45 years, n = 12) or a Trained Group (TG, 9.42 ± 0.51 years, n = 12). The intervention lasted eight weeks with a pre/post measurement (Δ) design in each group. It consisted of a combined cognitive and motor program composed of two 45 min sessions per week in TG. Reading and writing capabilities were measured using the word reading task based on the French battery (BALE) and the word dictation task, inspired by the ODÉDYS-2 French battery, respectively. The visuospatial orientation and the upper limb coordination parameters were evaluated using the Judgment of Line Orientation Test and Bruininks–Oseretsky test of motor proficiency, second edition, short form test, respectively. Results: Two-way ANOVA for repeated measures showed no significant difference between CG and TG in pre-intervention in all tests. Reading (*p* < 0.001, d = 1.19, Δ% = 15.07) and writing (*p* < 0.001, d = 1.13, Δ% = 19.69) scores increased significantly at post-compared to preintervention in the TG group. Comparable results were obtained in the visuospatial orientation (*p* < 0.001, d = 0.97, Δ% = 63.50) and the upper limb coordination (*p* < 0.001, d = 0.69, Δ% = 110.42) scores. No significant change was observed in CG comparing pre/post-intervention. Conclusions: A combined cognitive and motor training program could allow better cerebellar integration, leading to the improvement in reading, writing, and motor abilities in children with dyslexia. Further studies on a larger number of dyslexic children will be necessary to explore such issues.

## 1. Introduction

Developmental dyslexia (DD) is a specified learning disorder that is unrelated to abnormal intelligence, inadequate education, or sensory impairments such as visual or auditory deficit [1]. Dyslexics often struggle with accurate and/or fluent word recognition, along with significant difficulties in spelling and decoding [2]. According to a recent analysis by America’s Children and the Environment (ACE), 7.9% of children and adolescents aged 5 to 17 who were studied worldwide between 1997 and 2021 had DD [3]. Dyslexia’s origin remains not well defined; it is a multifactorial reading disorder, including genetic and environmental factors [4]. Researchers have proposed various theories to explain dyslexia. Several authors have consistently considered phonological deficits as the core cause of dyslexia [5,6,7]. This phonological theory suggests that children with dyslexia struggle to learn to read because they are unable to separate the sounds in words and associate them with their corresponding visual letter forms [8]. Current imaging-based studies have highlighted significant structural abnormalities in both cerebral connectivity and cortical structure, particularly in the left hemisphere language network, as underlying factors in the phonological deficits observed in dyslexia [9,10]. Overall, these results imply that the phonological theory emphasizes deficient phonological awareness but seems to be insufficient to explain all the deficits associated with DD [8]. Alternative theories have also been proposed, including auditory, visual perception, working memory, and attentional abnormalities [11,12,13,14,15].

A 2017 study by Le Floch and Ropars [16] highlighted visual abnormalities in individuals with dyslexia, specifically focusing on poor asymmetry between the two foveas (centroid of Maxwell’s spot), which appears to play a critical role in brain connectivity during typical child development. However, Stein [8] suggested that dysfunction in the dorsal stream might be a factor, but many researchers do not support this hypothesis, and the presence of a pathway dorsal deficit in dyslexia remains controversial [17]. Prompted by the challenges dyslexic readers face in attaining rapid and fluent reading, Nicolson and Fawcett [18,19] introduced the cerebellar theory of dyslexia. They proposed that cerebellar dysfunction is a fundamental factor in the development of dyslexia, causing a deficit in procedural learning. This deficit may explain reading difficulties in dyslexia as well as other related symptoms such as writing difficulties [20], spelling struggles, and spelling difficulties that are slow in handwriting [21,22], or motor problems [23,24]. At the same time, it is well known the poor motor abilities in dyslexic subjects are most likely related to poor cerebellum activities [25,26,27,28,29,30]. The cerebellar deficit theory, supported by various research findings, proposes that DD results from disrupted information processing due to mild neurobiological impairments in the cerebellum [18]. Certainly, structural differences in the cerebellum have been observed in individuals with dyslexia (e.g., [31]), including reduced cerebellar asymmetry compared to typically developing controls, who often exhibit a rightward cerebellar asymmetry [32,33,34]. Indeed, a study combining multiple imaging techniques (e.g., functional magnetic resonance imaging, functional, and structural connectivity) showed that difficulties in phoneme discrimination, a key feature of cerebral dysfunction in dyslexia, are linked to an inability to access intact phonemic representations through the subcortical white matter pathways, including the “arcuate fasciculus”, which connects Broca’s area to the temporo-parietal regions [35]. This finding directly implies that, unlike the majority of remediations currently employed with these children, dyslexia rehabilitation techniques should also focus on restoring functional connections between frontal and temporal language areas. Increasing the integration of information that is normally processed by various brain regions should be the main aim of rehabilitation [36]. Combined cognitive and motor training could be considered a valuable intervention to achieve this aim. The cerebellum, traditionally known for its role in motor control, is increasingly recognized for its involvement in cognitive processing such as reading and writing [37,38]. Cerebral plasticity refers to the ability of cerebral neural circuits to adapt and reorganize in response to experience, learning, and rehabilitation [39]. Thus, interventions such as combined cognitive and motor training could allow rhythmic stimulation, which enhances cerebral plasticity. One of the primary aims of research involving dyslexic children is to design training programs that can effectively enhance their reading abilities [36,40,41]. While linguistic training is the most studied [42], research has also explored various non-linguistic training methods such as motor training for improving both motor control and reading capabilities. First, Habib et al. [36], in order to test the efficacy of a specially designed cognitive-musical training (CMT) method, carried out the following two distinct studies: one in which dyslexic children underwent intensive musical exercises totaling 18 h over three consecutive days, and another in which the same 18 h of musical training were distributed across six weeks. Notable improvements were reported in both studies in various untrained, linguistic, and non-linguistic variables [36]. The first one produced significant improvement in auditory and categorical perception of speech’s temporal components [36]. Additional gains in reading comprehension, phonological awareness, auditory attention, and pseudo-word repetition were identified in the second study [36]. It is noteworthy that the majority of the benefits remained after an untrained period of six weeks. Ramezani and Fawcett [43] reported reading enhancement in children with dyslexia after fifteen training sessions (i.e., 3 days/week, 1 session/day, and 45–60 min/session). In this study [43], authors assessed the short-term impact of the dual-task Verbal Working Memory-Balance (VWM-B) program training on executive functions related to reading, reading skills, and reading comprehension in Persian children with DD [43]. However, to the best of the authors’ knowledge, no study investigated both reading and writing skills through a combined cognitive-motor intervention. Furthermore, our study presents a novel approach by highlighting the importance of motor coordination in reading and writing improvement in dyslexic children. This approach differs from existing training programs that target only language abilities. 

According to all the mentioned findings, the aim of the present study was to verify if an intervention based on a cognitive and motor program training could improve reading, writing, and motor coordination in Tunisian dyslexic children. Our driving hypothesis was that such intervention could allow better performance in cerebellar integration, via brain plasticity leading to a significant improvement in both cognitive and motor abilities with respect to a comparable group of dyslexic children who underwent school only.

## 2. Materials and Methods

### 2.1. Sample Size and Participants

The minimum required sample size was determined using the G*power software (version 3.1.9.6; Kiel University, Kiel, Germany). Based on prior research [44], the α and power (1 − β) values were set at 0.05 and 0.8, respectively. To ensure sufficient statistical power and minimize the risk of a type 2 error, data from twenty-four participants were deemed necessary.

Twenty-four children with dyslexia participated in this study and were randomly allocated to either a Control Group (CG, 9.25 ± 0.45 years, range: 9 to 10 years, n = 12) or a Trained Group (TG, 9.42 ± 0.51 years, range: 9 to 10 years, n = 12). All children were recruited from a special education and rehabilitation center of neuronal dysfunction pathologies in Tunisia. They were previously diagnosed as dyslexic by a licensed educational psychologist according to the Diagnostic and Statistical Manual of Mental Disorder-IV Criteria, including abnormal reading performances and poor writing abilities compared to their expected level for chronological age [45]. Clinical characteristics of the participants are shown in Table 1.

The inclusion criteria were no history of vestibular, orthopedic, neurological, or psychiatric pathology; normal motor development; normal mean intelligence quotient (evaluated with Wechsler Intelligence Scale for Children, Fourth Edition, WISC-IV [46]; and dyslexia. Non-inclusion criteria were any known comorbidities, vestibular disorder, orthopedic disorder, intellectual disability, chronic medical disorders, visual and/or physical impairments, neurological disorders (i.e., subjects with cerebral palsy, epilepsy, neuromuscular disorders, and tethered spinal cord), and a significant discontinuity in their schooling.

**Figure 1:**
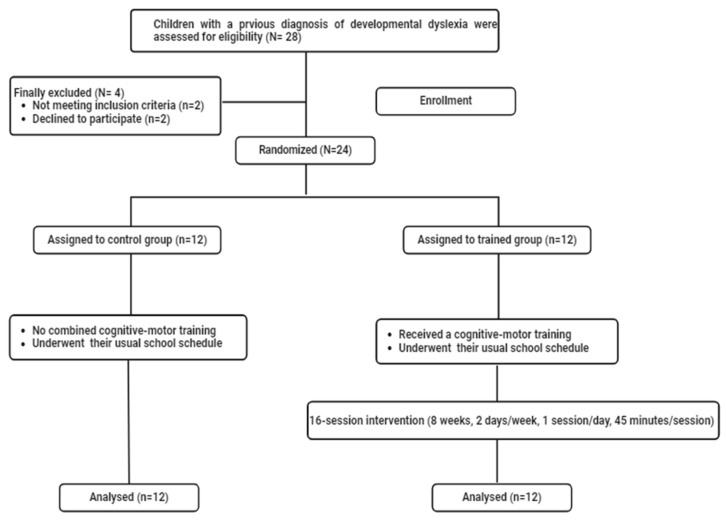
Flowchart of participants’ recruitment.

During the intervention period, all children did not attend any additional exercise training in or out of school. They were asked to maintain their habitual rhythm of being asleep/awake. Oral and written consent were obtained from all children and their legal guardians, respectively. Before, the experimental procedure was explained to them. Figure 1 shows the participants’ recruitment flow chart.

The present study was conducted in accordance with the Declaration of Helsinki and was approved by the Ethics and Research Committee (IORG 0007439 ERC04042024).

### 2.2. Cognitive Evaluation

#### 2.2.1. Reading Evaluation

Reading ability was evaluated for each child using the reading subtest inspired by the French battery (Batterie Analytique du Langage Ecrit, BALE) [47]. This test comprises the following two sets of items: high- and low-frequency words. Three lists of printed words were included in each set, grouped in the following columns: 20 regular words, 20 irregular words, and 20 pseudowords. The child had to read the words in each column as clearly as possible. The number of words correctly read was counted, and the success score was marked out of 20 for each column [47].

#### 2.2.2. Writing Evaluation

Writing skills were evaluated through the dictation test extract from (Outil de DÉpistage des DYSlexies Version 2, ODÉDYS 2) French battery, [48]. The ODÉDYS 2 was developed by Michèle Jacquier-Roux, Sylviane Valdois, and Michèle Zorman. It was published by the Cogni-Sciences Laboratory, located in Grenoble, France. This test allows you to explore the writing abilities of each child, as well as the orthographic production. During the task, three lists of 10 words, totaling 30 words, were proposed for the child that corresponded to the following three categories of words: regular words, irregular words, and pseudo-words. The child was asked to write the dictated words in columns and note the score, out of 10, of correctly spelled words for each word category [48].

#### 2.2.3. Combined Cognitive and Motor Training Program

The combined cognitive and motor training program consisted of two training sessions per week for eight weeks. Each training session lasted 45 min and was composed of the following four different parts: 10 min was allocated to warming up (light movements to activate upper limbs and improve spatial awareness), followed by 30 min of both motor and cognitive exercises. The last five minutes were dedicated to cooling down the body and stretching. Motor and cognitive exercises were composed of two levels of difficulty (each of them lasted four weeks; see Table 2).

### 2.3. Motor Evaluation

#### 2.3.1. Visuospatial Orientation Evaluation

The visuospatial parameter was measured using the Judgment of Line Orientation Test (JLOT), which was developed to evaluate the visuospatial abilities [49]. The test includes a spiral booklet consisting of 35 pages with an array of 11 lines, each drawn by 18° angles. The participants were instructed to match the pair of lines on each page with those 11 lines by visually estimating their angles. Consistent with test procedures, both stimulus lines must be correctly identified in order to receive a raw score of one for each item (total possible score = 30 points). A raw score of zero for an item was given when either one or none of the stimulus lines in the item was correctly identified [50].

#### 2.3.2. Upper Limb Coordination Evaluation

Bruininks–Oseretsky Test of Motor Proficiency, Second Edition, Short Form (BOT-2 SF; [51]) was used to measure the upper limb coordination parameter. This test allows measuring fine and gross motor abilities development in children aged from four to 21 years old [52]. The BOT-2 SF comprises 14 items grouped under the following eight distinct motor proficiency subtests: fine motor precision, fine motor integration, manual dexterity, bilateral coordination, balance running speed and agility, upper limb coordination, and strength. For the present study, only the upper limb coordination subtest was assessed based on two items (the dribbling ball-alternating hands and the dropping and catching a ball) in order to measure visual tracking with coordinated arm and hand movement for each child.

During the dribbling ball-alternating hands item, the child was asked to dribble a ball (i.e., a tennis ball) using both hands alternately. Scoring for this task typically involves assessing the child’s ability to maintain a continuous and controlled dribbling pattern. The experimenter counted the number of correct dribbles up to a maximum of 10. A dribble is incorrect if the child did not alternate hands with each dribble, caught the ball, or let the ball bounce more than once between dribbles. During the dropping and catching a ball item, the child drops the ball on the ground and after the bounce and then catches it with both hands. The experimenter recorded the number of successful caches up, with a maximum score of five. A catch is incorrect if the subject pins the ball against the chest or catches it with one hand. For each item, the point scores were summed, creating total point scores.

Before training started, the children of TG were familiarized with the experimenter, material/devices, and exercises used during the training procedure.

### 2.4. Experimental Procedure

Children of the TG underwent the regular school schedule but also had 16 training sessions for eight weeks, whereas children of the CG conducted the regular school schedule only without participating in any training program. At T0 (before the 8-week intervention) and at T1 (after the 8-week intervention), we measured both cognitive and motor parameters. For the cognitive evaluation, we used a word reading task based on the French battery, BALE [47], to measure reading capabilities. Furthermore, we evaluated writing capabilities through a word dictation task, inspired by the ODÉDYS 2 French battery [48]. In order to evaluate motor parameters, we used JLOT [49] to evaluate the visuospatial parameter and the dribbling ball-alternating hands subtest, inspired by BOT-2 SF [50], to evaluate the upper limb coordination.

Before and after the training program, the TG and the pre- and post-testing sessions in both reading and writing capabilities were assessed two times, at T0 and T1, respectively, before and after the training program in the TG and before and after eight weeks without any training in CG.

### 2.5. Data Analysis

During reading and writing tests, we measured the number of correct words read, and we calculated the number of correct words written by each child using the BALE and the ODÉDYS 2 French batteries [47,48].

For the JLOT [49] test, the number of correct items was the variable measured that is related to the visuospatial capabilities. In the BOT-2 SF [51], the count of the number of ball dribbles in the first item and the number of catches in the second is able to give an insight into the upper limb coordination. All these variables measured at T0 and T1 were compared and underwent statistical analysis.

### 2.6. Statistical Analysis

Analyses were performed using Excel (Microsoft Office, v. 2016) and SPSS Statistics (IBM, v.21, SPSS Inc., Armonk, NY, USA) software. All the data, either for cognitive (i.e., number of correct words read and written during BALE and the ODÉDYS 2) or motor variables (i.e., number of correct items reported during JLOT and the number of ball dribbling and ball catches measured during the BOT-2 SF subtest), were expressed as means ± standard deviations (SD).

Normality was checked using the Shapiro–Wilk W-test. For both groups, values of respect and significance are well above the significance threshold (*p* = 0.05). This leads to the conclusion that the data were normal. The analysis was performed between the two groups of dyslexic children using a two-way repeated measures ANOVA [2 conditions (TG and CG) ×2 times (T0 and T1)]. When significant main or interaction effects were observed, the Bonferroni post-hoc test was conducted. Significance was considered when the *p*-value was below 0.05. The ANOVA effect sizes were calculated as partial eta squared (ɳp^2^), and values of 0.01, 0.06, and 0.13 represented small, moderate, and large effect sizes, respectively [53]. In addition, the effect size (Cohen’s d) of pairwise analysis was calculated using the following thresholds: <0.20 (trivial), 0.20–0.60 (small), 0.60–1.20 (moderate), 1.20–2.0 (large), 2.–4.0 (very large), and >4.0 (extremely large) [54].

## 3. Results

The mean ± SD of cognitive and motor parameters measured in our population of dyslexic children before the intervention compared to typical non-dyslexic children is displayed in Table 3.

Regarding normal values in non-dyslexic children, we referred to Jacquier-Roux et al. [47,48] for reading and writing scores, Mersin and Çebi [55] for visuospatial scores, and Alsaedi [56] for upper limb coordination scores.

### 3.1. Cognitive Abilities

Mean ± SD values of cognitive parameters for both groups of children are shown in Table 4. It should be noted that the measurement instruments used in the present study aimed to evaluate reading speed and accuracy based on the BALE test. Moreover, the ODÉDYS 2 dictation test was used to measure writing fluency and lexical spelling.

#### 3.1.1. Reading Test

**Figure 2:**
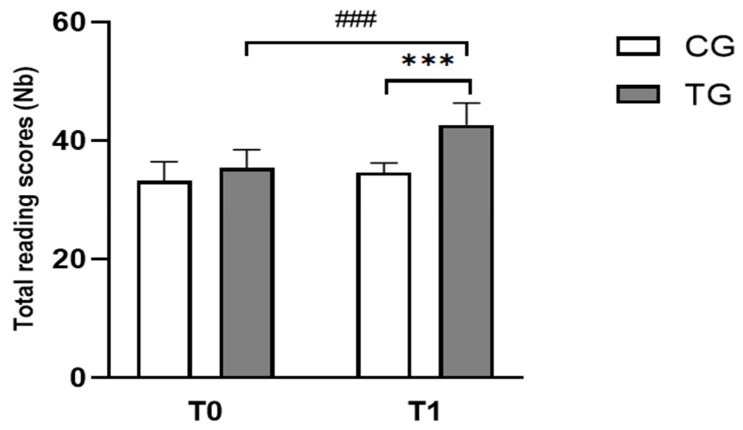
Mean ± standard deviation values of the number of words read during the BALE reading subtest for the two groups of dyslexic children at T0 and T1. CG: Control Group; TG: Trained Group; T0: Before the intervention; T1: After the intervention. ^###^ *p* < 0.001, comparison between pre- and post-intervention in TG; *** *p* < 0.001, comparison between TG and CG in post-intervention.

Repeated measure ANOVA revealed significant effects of time (F_(1,22)_ = 61.00, *p* < 0.001, ɳp^2^ = 0.73), group (F_(1,22)_ = 17.29, *p* < 0.001, ɳp^2^ = 0.44), and (group × time) interaction (F_(1,22)_ = 20.08, *p* < 0.001, ɳp^2^ = 0.47). The Bonferroni post-hoc test showed that reading scores were significantly higher after program intervention (T1) compared to T0 in TG (*p* < 0.001, d = 1.19, Δ% = 15.07; Figure 2). Moreover, these scores were higher in TG compared to CG at T1 (*p* < 0.001, d = 1.30, Δ% = 21.46; Figure 2).

#### 3.1.2. Writing Test

**Figure 3:**
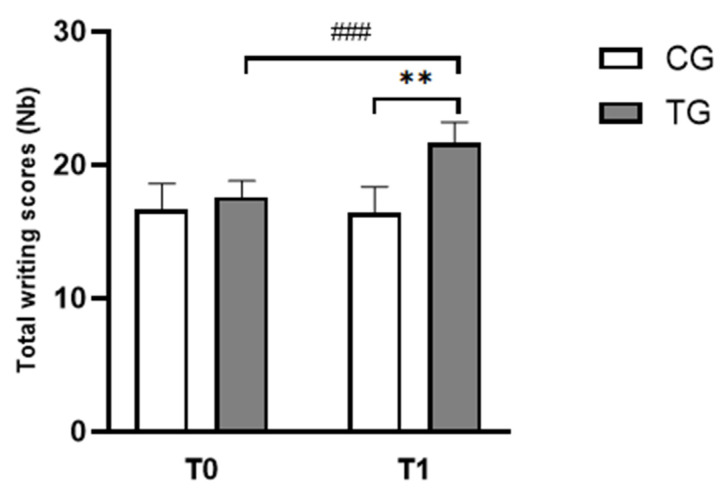
Mean ± standard deviation values of the number of correct words during the ODÉDYS 2 writing subtest for the CG and EG before and after the training program recorded at T0 and T1. CG: Control Group; TG: Trained Group; T0: Before the intervention; T1: After the intervention. ^###^ *p* < 0.001, comparison between pre- and post-intervention in TG; ** *p* < 0.01, comparison between TG and CG in post-intervention.

Figure 3 summarizes the scores measured in the two groups measured at T0 and T1. ANOVA revealed significant effects of time (F_(1,22)_ = 102.09, *p <* 0.001, ɳp^2^ = 0.82), group (F_(1,22)_ = 10.04, *p* = 0.004 and ɳp^2^ = 0.31) and (group × time) interaction (F_(1,22)_ = 50.41, *p* < 0.001, ɳp^2^ = 0.69). The Bonferroni post-hoc test revealed that scores of correct words during the writing task increased significantly in TG at T1 compared to T0 (*p* < 0.001, d = 1.13, Δ% = 19.69; Figure 3). In addition, TG showed higher writing scores compared to those in CG (*p* < 0.01, d = 1.84, Δ% = 23.86; Figure 3).

### 3.2. Motor Abilities

Mean ± SD values related to motor parameters in each group are displayed in Table 5.

#### 3.2.1. Visuospatial Orientation Test

**Figure 4:**
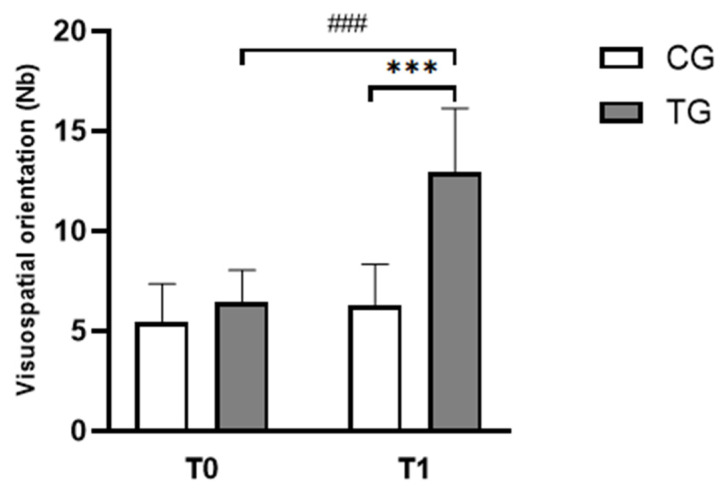
Mean ± standard deviation values of total scores during the JLOT task for both groups in T0 and T1 sessions. CG: Control Group; TG: Trained Group; T0: Before the intervention; T1: After the intervention. ^###^ *p* < 0.001, comparison between pre- and post-intervention in TG; *** *p* < 0.001, comparison between TG and CG in post-intervention.

Significant effects of time (F_(1,22)_ = 53.95, *p* < 0.001, ɳp^2^ = 0.71), group (F_(1,22)_ = 23.21, *p* < 0.001, ɳp^2^ = 0.51), and (group × time) interaction (F_(1,22)_ = 32.02, *p* < 0.001, ɳp^2^ = 0.60) were observed. The Bonferroni post-hoc test showed that the JLOT scores increased significantly at T1 compared to T0 in TG group (*p* < 0.001, d = 0.97, Δ% = 63.50; Figure 4). Although, during the post-intervention test, performances were higher in the TG compared to the CG group (*p* < 0.001, d = 1.19, Δ% = 134.32; Figure 4).

#### 3.2.2. Upper Limb Coordination Test

**Figure 5:**
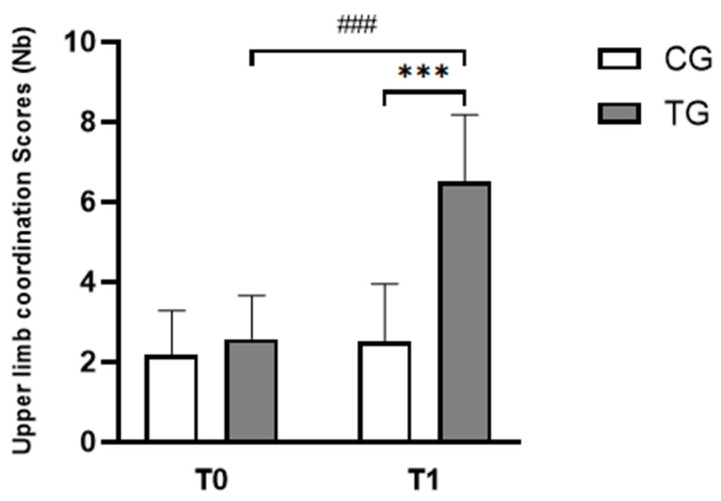
Mean ± standard deviation values of the number of ball dribbles and catches during the BOT-2 SF subtest before and after the training program across the different groups. CG: Control Group; TG: Trained Group; T0: Before the intervention; T1: After the intervention. ^###^ *p* < 0.001, comparison between pre- and post-intervention in TG; *** *p* < 0.001, comparison between TG and CG in post-intervention.

Scores of the BOT-2 SF subtest are shown in Figure 5. The two-way repeated measure ANOVA test revealed significant effects of time (F_(1,22)_ = 57.33, *p* < 0.001, ɳp^2^ = 0.72), group (F_(1,22)_ = 21.53, *p* < 0.001, ɳp^2^ = 0.49), and (group × time) interaction (F_(1,22)_ = 40.76, *p* < 0.001, ɳp^2^ = 0.65). The Bonferroni post-hoc test revealed that values of BOT-2 SF scores were significantly higher at T1 compared to T0 in the TG group (*p* < 0.001, d = 0.69, Δ% = 110.42; Figure 5). Furthermore, higher performances were observed in TG compared to CG (*p* < 0.001, d = 1.30, Δ% = 250.42; Figure 5).

## 4. Discussion

The main findings of our study were the improvements of both reading and writing capabilities and motor coordination in dyslexic children as consequences of an 8-week combined cognitive and motor program training.

### 4.1. Cognitive Performances

Our results revealed that dyslexic children exhibited better performances in reading and writing capabilities after the cognitive-motor training program in comparison to the CG, who underwent school only. According to Caldani et al. [38], the beneficial effects of cognitive-motor training on the reading abilities of dyslexic children were investigated [38]. In more detail, these authors evaluated reading speed after a vestibular and cognitive training program in nineteen children with dyslexia (9.48 ± 0.15 years) after a 4-week training program. Training consisted of four exercises conducted on a Wacom tablet, performed for 16 min per session, twice a week, over four weeks. Each exercise consisted of eight levels with progressively increasing difficulty [38]. Reading speed was evaluated using the ELFE French reading test (Évaluation de la Lecture en FluencE) [57]. The authors reported a significant improvement in reading speed after the vestibular and cognitive training, suggesting the improvement in the vestibular network leading to reading enhancement in dyslexic children. However, the study by Caldani et al. [38] focused only on the reading speed, suggesting that such a type of vestibular and cognitive training, based on a tablet, could be beneficial for improving reading performance in children with dyslexia. Furthermore, it is well documented that cognitive deficits in dyslexic children are related to poor cerebellar function [18,19,31,32,33,34]. Consequently, and according to these previous studies, the improvements observed in the present study could be explained by an implicit increase in participants’ cerebellar activity and central plasticity. This hypothesis is supported by the results of a more recent study conducted by Ramezani and Fawcett [40], showing that cognitive-motor training, based on a dual-task Verbal Working Memory-Balance (VWM-B) program (15 session intervention, three days/week, one session/day, 45–60 min/session), could increase activation of specific cerebral or cerebellar regions in trained dyslexic children compared to their controls who received training using the single-task Verbal Working Memory (VWM) program. In this 2024 study of Ramezani and Fawcett [40], an enhancement in cognitive and motor abilities was observed, underscoring the significance of the cerebellum’s role in this disorder. Accordingly, the VWM-B program activates critical cerebral and cerebellar regions, which are essential for various reading skills [58,59]. Future studies can explore the possible association between the implicit activation of cerebral regions induced by other types of motor-cognitive programs, highlighting its effect on both reading and writing capabilities, which is the case in our study. Next, we will focus on the discussion of findings observed in dyslexic children (between T0 and T1) and their difference with respect to typical non-dyslexic children. It is likely that before the combined cognitive-motor program intervention (T0), cognitive parameters measured in dyslexic children of the present study were lower compared to non-dyslexic children (Table 3). Nevertheless, our results revealed that despite the improvement shown in trained dyslexic children of the present study after the intervention (T1), values remained lower than the known norms in non-dyslexic children. Based on the BALE [47] and ODÉDYS 2 [48] scores, trained dyslexic children still had abnormal reading and writing levels compared to normal children in the same age. Thus, these differences shown in trained children compared to typical non-dyslexic children may be explained by dyslexia severity. The children recruited in the present study might have severe forms of dyslexia, which could result in slower or incomplete improvements compared to expected norms, even with effective combined cognitive-motor training. Moreover, it is suggested that our findings are limited by partial brain plasticity and/or the duration of the program.

### 4.2. Motor Performances

The TG showed a significant improvement in visuospatial abilities after the program intervention compared to the CG. However, the TG made more ball dribbles during the upper limb coordination task compared to their controls. Otherwise, the novelty of the cognitive and motor training used in our study is that we decided to improve both reading and writing capabilities through improving motor coordination in dyslexic children by improving specific motor components (upper limb coordination and visuospatial orientation), which present motor deficits in these subjects.

Researchers frequently highlight visuospatial competency as a critical factor in dealing with issues related to both reading [60] and writing skills [61,62]. Nevertheless, the ability to recognize size and shape constancy, along with an understanding of depth and spatial awareness, should develop during early childhood [63]. Unfortunately, perturbation in this competency can lead to later challenges with letter identification, memorizing their sequences within words, and related tasks [64]. In previous literature, such difficulties are referred to as visuospatial deficits [65], such challenges are frequently observed in children with dyslexia [66]. Dyslexic children often struggle to orient their bodies in space, to organize objects, and to represent shapes. These difficulties extend to orienting letter shapes, memorizing the direction of past words, following present ones, and anticipating the future flow of text, something frequently experienced by dyslexics [64]. Therefore, we designed in the present study a structured combined training program, which aims to address this motor deficit observed in dyslexic children. In other words, we suggest that if the child could distinguish the correct orientation of the different letters, especially of those which may be a source of confusion for him (e.g., “b/d” or “m/n” or “p/q”), he could achieve better reading and writing performances.

However, a previous study revealed [67] that dyslexia has been associated through the literature with other motor impairments such as upper limb coordination deficits. Dyslexics suffer not only from reading and writing disabilities but also more coordination deficits can be observed in this population [67]. In a cross-sectional study conducted on 200 children (i.e., 100 typical and 100 with specific learning disabilities), Hussein et al. [68] reported that 100% of dyslexic children struggle with upper limb coordination. Accordingly, results of previous research show that motor disabilities of upper limb coordination of children among the examined population are in direct correlation with dyslexia [69].

Furthermore, it is suggested that the improvement in motor deficit through a training intervention could be useful to help dyslexic children to write letters in the correct way and to follow words’ fluency during reading tasks. Taken together, it is likely that in order to achieve good writing and reading performances, a good control of upper limb coordination is necessary in dyslexic children. This motor function seems to play a critical role in both processes, according to Macdonald et al. [70], who identified upper limb coordination as the component of gross motor proficiency and linked it to reading performances. In the same line, results of a systematic review [71] revealed a significant correlation between upper limb coordination and reading ability across various age groups, including kindergarten children [72], students 5 years [73], and adolescents [74].

Moreover, our results are in line with those of previous studies highlighting the fact that efficient upper limb coordination is useful to perform better writing capabilities. According to the study by Nilukshika et al. [75] conducted on 40 undergraduate students and including a structured motor exercise program for four weeks (5 days/week; 20 min/day), aiming to strengthen the muscles involved in handwriting [75]. Results showed that writing skills could be improved through the implementation of upper limb motor programs [75].

Finally, the same findings already shown in cognitive parameters were observed in motor parameters when comparing our results in T0 to normal values in non-dyslexic children (Table 3). After the training intervention (T1), lower performances in JLOT and BOT-2 scores were observed in TG compared to typical non-dyslexic children [55,56]. Consistently, we hypothesized that the duration of our intervention may not have been sufficient to produce long-lasting and significant changes relative to normal levels. The cognitive-motor training program used in this study lasted only eight weeks, suggesting that longer training periods may be necessary to achieve more noticeable improvements.

### 4.3. Strengths and Limitations

This study offers novel insights into the comprehension and the effectiveness of the combination of both cognitive and motor training in dyslexic children. While this study presents valuable insights, there are some limitations to note. Our training program was based only on two motor parameters (i.e., upper limb coordination and visuospatial abilities), providing an impetus for conducting future randomized trials while controlling other motor parameters. Additionally, the participants’ number was modest, which may influence the generalizability of our findings. Lastly, future studies could consider incorporating a more precise evaluation that engages measurement instruments, allowing us to determine whether the observed improvements can be attributed to other specific reading and writing elements.

## 5. Conclusions

The present study revealed notable enhancement in reading and writing capabilities and motor coordination because of the combined motor-cognitive training in dyslexic children. The observed positive changes underscore the potential role of the combined motor and cognitive training in improving both writing and reading capabilities in this population. Furthermore, our results highlighted the impact of the improved motor parameters on cognitive capabilities. Finally, we suggest that such intervention can be used in rehabilitation activities in this population. Nevertheless, including an objective measurement of cerebral activity can provide valuable insights for future studies.

## Figures and Tables

**Table 1:** Mean ± standard deviation (SD) values of clinical characteristics of both groups of dyslexic children.

Clinical Characteristics	Mean (±SD)	
	CG (n = 12)	TG (n = 12)
Girls/boys (Nb)	6/6	6/6
Chronological age (years)	9.25 ± 0.45	9.42 ± 0.51
Weight (kg)	30.08 ± 1.65	30.33 ± 1.63
Height (cm)	130.67 ± 2.02	130.58 ± 1.73
IQ (WISC-IV)	103.92 ± 5.20	104.42 ± 4.19

CG: Control Group; TG: Trained Group; IQ, intelligence quotient; WISC-IV, Wechsler Intelligence Scale for Children—Fourth Edition.

**Table 2:** Exercises proposed through the cognitive and motor training program.

Activity Stage	Performed Exercises
Warm-Up (10 min)	Arm circles (forward/backward)
	Cross-body arm swings
	Shoulder rolls
Upper limb coordination (15 min)	1. Ball Toss and Call-Out (5 min)
	Toss a ball against a wall or with a partner, catching it with one or both hands.
	2. Ball Passing (5 min)
	Pass a ball from one hand to the other while calling out confusion’s letters (e.g., “b/d” or “m/n” or “p/q”).
	3. Handball Juggling (5 min)
	Hold a tennis ball in each hand and throw them in the air alternately, catching them with the same hand. Gradually increase the height and speed.
Visuospatial orientation (15 min)	1. Direction Changes (5 min)
	Change direction through different orientations (e.g., “left” or “right” or “forward” or “backward”) according to verbal or visual instructions.
	2. Draw and Follow (5 min)
	Draw a line representation of confusions’ letters (e.g., “b/d” or “m/n” or “p/q”). Follow the representation’s direction to reach the destination.
	Ball dribbling followed a path drawn that represented confusion among letters (e.g., “b/d” or “m/n” or “p/q”).
	3. Mirror Exercises (5 min)
	A partner calls out or demonstrates movements (e.g., “step right and turn left”) while you mirror them.
Cool-Down (5 min)	Stretch arms overhead and across the chest.
	Deep breathing with arm raises.

**Table 3:** Mean ± standard deviation (SD) of cognitive and motor parameters measured in the 24 dyslexic children of the present study before the intervention compared to non-dyslexic children.

Cognitive and Motor Parameters	Participants of this Study	Non-Dyslexic Population
Reading scores (Nb)	33.96 ± 2.60	55.19 ± 4.42 [47]
Writing scores (Nb)	16.54 ± 1.89	26.28 ± 3.65 [48]
Visuospatial orientation scores (Nb)	5.92 ± 1.82	21.60 ± 2.89 [55]
Upper limb coordination scores (Nb)	2.38 ± 1.10	10.20 ± 1.73 [56]

**Table 4:** Mean ± standard deviation (SD) values of cognitive parameters for each group (CG and TG) at T0 and T1.

Cognitive Parameters	Groups	Means Values (±SD)	
		T0	T1
Reading scores (Nb)	CG	33.25 ± 3.22	35.42 ± 3.09
	TG	34.67 ± 1.61	42.67 ± 3.70 ^###^
Writing scores (Nb)	CG	16.67 ± 1.92	17.58 ± 1.24
	TG	16.42 ± 1.93	21.67 ± 1.44 ^###^

T0: Before the intervention; T1: After the intervention; CG: Control Group; TG: Trained Group; Significant difference between T0 and T1 in TG: ^###^ *p* < 0.001.

**Table 5:** Mean ± standard deviation (SD) of motor parameters (spatial orientation and upper limb coordination) for the two groups (CG and TG) at T0 and T1.

Motor Parameters	Groups	Means Values (±SD)	
		T0	T1
Visuospatial orientation scores (Nb)	CG	5.42 ± 1.93	6.25 ± 2.09
	TG	6.42 ± 1.62	12.92 ± 3.23 ^###^
Upper limb coordination scores (Nb)	CG	2.17 ± 1.11	2.50 ± 1.45
	TG	2.58 ± 1.08	6.50 ± 1.68 ^###^

T0: Before the intervention; T1: After the intervention; CG: Control Group; TG: Trained Group; Significant difference between T0 and T1 in TG: ^###^ *p* < 0.001.

## Data Availability

The original contributions presented in this study are included in the manuscript; further inquiries can be directed to the corresponding authors.

## Abbreviations

The following abbreviations are used in this manuscript:

ACE	America’s Children and the Environment
DD	Developmental Dyslexia
BALE	Batterie Analytique du Langage Ecrit
BOT-2 SF	Bruininks–Oseretsky Test of Motor Proficiency, Second Edition, Short Form
ELFE	Évaluation de la Lecture en FluencE
JLOT	Judgment of Line Orientation Test
ODÉDYS 2	Outil de DÉpistage des DYSlexies Version 2
VWM	Verbal Working Memory
VWM-B	Verbal Working Memory-Balance
WISC-IV	Wechsler Intelligence Scale for Children, Fourth Edition
